# Protective Effects of α-Tocopherol, γ-Tocopherol and Oleic Acid, Three Compounds of Olive Oils, and No Effect of Trolox, on 7-Ketocholesterol-Induced Mitochondrial and Peroxisomal Dysfunction in Microglial BV-2 Cells

**DOI:** 10.3390/ijms17121973

**Published:** 2016-11-25

**Authors:** Meryam Debbabi, Thomas Nury, Amira Zarrouk, Nadia Mekahli, Maryem Bezine, Randa Sghaier, Stéphane Grégoire, Lucy Martine, Philippe Durand, Emmanuelle Camus, Anne Vejux, Aymen Jabrane, Lionel Bretillon, Michel Prost, Thibault Moreau, Sofien Ben Ammou, Mohamed Hammami, Gérard Lizard

**Affiliations:** 1 Team ‘Biochemistry of the Peroxisome, Inflammation and Lipid Metabolism’ EA 7270/INSERM, University Bourgogne Franche-Comté, 21000 Dijon, France; debbabi.meryam55@gmail.com (M.D.); thomas.nury@u-bourgogne.fr (T.N.); zarroukamira@gmail.com (A.Z.); nadia.mekahli@yahoo.fr (N.M.); bezzinemaryem@yahoo.fr (M.B.); sg.randa@yahoo.fr (R.S.); anne.vejux@u-bourgogne.fr (A.V.); 2 Lab-NAFS ‘Nutrition—Functional Food & Vascular Health’, University Monastir, LR12ES05, 5000 Monastir, Tunisia; mohamed.hammami@fmm.rnu.tn; 3 Faculty of Medicine, University Sousse, 4000 Sousse, Tunisia; 4 Laboratory ‘Venoms & Therapeutic Biomolecules’, Pasteur Institute, University Tunis El Manar, 1000 Tunis, Tunisia; 5 Eye & Nutrition Research Group, CSGA, UMR 1324 INRA, 6265 CNRS, University Bourgogne Franche-Comté, 21000 Dijon, France; stephane.gregoire@dijon.inra.fr (S.G.); lucie.martine@dijon.inra.fr (L.M.); lionel.bretillon@dijon.inra.fr (L.B.); 6 Kirial International/Laboratoires Spiral, 21560 Couternon, France; p.guyondet.spiral@orange.fr (P.D.); camusprost@hotmail.fr (E.C.); michelprost.spiral@wanadoo.fr (M.P.); 7 Société Nopal Nutra, 21000 Dijon, France; aymen.jabrane@nopalnutra.com; 8 Department of Neurology, University Hospital of Dijon, 21000 Dijon, France; thibault.moreau@chu-dijon.fr; 9 Department of Neurology, University Hospital Sahloul, 4000 Sousse, Tunisia; sofien.benammou@rns.tn

**Keywords:** α-tocopherol, γ-tocopherol, Trolox, oleic acid, mitochondria, peroxisome, murine microglial BV-2 cells

## Abstract

Lipid peroxidation products, such as 7-ketocholesterol (7KC), may be increased in the body fluids and tissues of patients with neurodegenerative diseases and trigger microglial dysfunction involved in neurodegeneration. It is therefore important to identify synthetic and natural molecules able to impair the toxic effects of 7KC. We determined the impact of 7KC on murine microglial BV-2 cells, especially its ability to trigger mitochondrial and peroxisomal dysfunction, and evaluated the protective effects of α- and γ-tocopherol, Trolox, and oleic acid (OA). Multiple complementary chemical assays, flow cytometric and biochemical methods were used to evaluate the antioxidant and cytoprotective properties of these molecules. According to various complementary assays to estimate antioxidant activity, only α-, and γ-tocopherol, and Trolox had antioxidant properties. However, only α-tocopherol, γ-tocopherol and OA were able to impair 7KC-induced loss of mitochondrial transmembrane potential, which is associated with increased permeability to propidium iodide, an indicator of cell death. In addition, α-and γ-tocopherol, and OA were able to prevent the decrease in Abcd3 protein levels, which allows the measurement of peroxisomal mass, and in mRNA levels of Abcd1 and Abcd2, which encode for two transporters involved in peroxisomal β-oxidation. Thus, 7KC-induced side effects are associated with mitochondrial and peroxisomal dysfunction which can be inversed by natural compounds, thus supporting the hypothesis that the composition of the diet can act on the function of organelles involved in neurodegenerative diseases.

## 1. Introduction

In the ageing process and in numerous age-related diseases, including neurodegenerative diseases, mitochondrial dysfunction, disruption of RedOx status and increased production of reactive oxygen species (ROS) are considered key events [1,2,3,4]. In ageing, the decrease in telomere length, which is important in the control of longevity, is a result of the combined effects of oxidative stress and repeated cell replications [5]. FOXO transcription factors, which play important roles in the signal transduction pathway associated with stress and aging phenotypes, are also strongly regulated by oxidative stress [6]. In major demyelinating and non-demyelinating neurodegenerative diseases including Multiple Sclerosis (MS), X-linked adrenoleukodystrophy (X-ALD), Alzheimer’s disease, Parkinson’s disease and amyotrophic lateral sclerosis, oxidative stress is thought to contribute to neuronal loss and axonal damage, which are considered key elements in the onset and progression of these diseases [7].

Currently, in brain aging and neurodegeneration, the activation of microglial cells is believed to play simultaneous beneficial and detrimental roles [8,9]. In MS, microglial cells have been reported to exert detrimental effects on neurons and oligodendrocytes, including toxic effects through the release of proteases, the production of inflammatory cytokines, ROS and reactive nitrogen species (RNS), and the recruitment and reactivation of T lymphocytes [10]. However, beneficial roles have also been reported, including roles in axonal regeneration, the promotion of remyelination, the clearance of inhibitory myelin debris and the release of neurotrophic factors [10]. In Alzheimer’s disease, it is well established that microglial cells are associated with senile plaques and can contribute to the clearance of β-amyloid (Aβ) [11]. In Abcd1-deficient mice (*Abcd1*^−^; an X-ALD mouse model), astrocytosis and microgliosis, identified with glial fibrillary acidic protein (GFAP) and lectin staining, have been reported mainly in the pyramidal tracts and dorsal fascicles [12,13,14]. Data obtained on autopsy material of white matter from X-ALD patients with different forms of the disease and on the brain of mice injected with diC24:0 phosphatidylcholine in the area of the white matter of the corpus callosum support the hypothesis that microglial apoptosis in perilesional white matter represents an early stage in lesion evolution [15]. In X-ALD, based on data obtained from the brains of patients with the most common form of the disease (childhood adrenoleukodystrophy, cALD), which is often rapidly fatal, it has been suggested that very long chain fatty acid accumulation in membrane domains associated with signal transduction might trigger inflammatory processes through the activation of astrocytes and microglial cells [16]. In agreement with this hypothesis, activated microglia have been revealed by positron emission tomography in an X-ALD patient [17].

As it is assumed that oxidative stress contributes to neurodegeneration, it can be supposed that environmental factors coming from lipid peroxidation, such as aldehydes (resulting from fatty acid oxidation) and some oxysterols, such as 7-ketocholesterol (7KC) (mainly deriving from the auto-oxidation of cholesterol) [18,19,20], can cause brain damage and modulate microglial activity. Indeed, increased levels of 7KC have been reported in the plasma and/or the cerebrospinal fluid of patients with neurodegenerative diseases. Thus far, 7KC has been identified at increased levels in the cerebrospinal fluid of patients with MS [21] as well as in the plasma of patients with X-ALD [22] and Niemann–Pick disease [23]. 7KC has also been detected at higher levels in the plasma of patients with the progressive form of MS than in plasma from patients with the relapsing remitting form of MS [24]. On the other hand, 7KC could render the plasma membrane more sensitive to Aβ-induced neurotoxicity [25]. As microglial cells are the primary immune cells of the central nervous system and are extremely similar to monocytes/macrophages, which are activated in the presence of 7KC (induction of oxidative stress (H_2_O_2_ and O_2_^•−^ overproduction, lipid peroxidation, 8-oxoguanine formation) and cytokine secretion (IL-1β, IL-8, MIP-1β) leading to cell death at elevated concentrations [26,27,28], it is tempting to speculate that 7KC might also trigger microglial activation and/or dysfunction (rupture of RedOx homeostasis and inflammation: ROS, RNS and cytokines overproduction, increased phagocytosis) associated with alterations of major organelles (mitochondria, peroxisome) known to contribute to the development of several degenerative diseases.

Currently, the impact of 7KC on mitochondrial dysfunction, which is often observed in neurodegeneration, is well documented [29,30]. However, in neurodegenerative diseases (except for peroxisomal leukodystrophies [3]), the role played by the peroxisome, which is tightly connected with mitochondria and can affect RedOx-linked physiological processes [31,32], is still not well known. Far less is known about the role of peroxisomes in ageing and cell death than is the case for the role of mitochondria. It is, however, now conceivable that mitochondrial dysfunction can be linked to the peroxisomal status [33]. In BV-2 murine microglial cells, there is some evidence that 7KC-induced cell death simultaneously triggers mitochondrial and peroxisomal dysfunction [22].

In addition, since oxidative stress may be one of the first and major hits in neurodegeneration, it would be useful to identify molecules able to cross the blood-brain barrier and to attenuate microglial activation resulting from disturbance of the extracellular and/or intracellular RedOx status under the action of lipid peroxidation products, such as 7KC. At the moment, only a few natural (α-tocopherol, docosahexaenoic acid, polyphenols) and synthetic (dimethylfumarate) molecules are able to protect against the toxic effects of 7KC as described below. Among these molecules, α-tocopherol is one of the most effective in various cell types, including nerve cells [27,34,35,36]. Its main function is to trap lipid peroxyl radicals (LOO^•^) by preventing the propagation of lipid peroxidation [37,38,39]. α-Tocopherol is known to prevent 7KC-induced loss of mitochondrial transmembrane potential in numerous cell types and to counteract the accumulation of 7KC in lipid rafts [34,40,41]. These effects of α-tocopherol were not observed with γ-tocopherol in A7R5 rat smooth muscle cells [41]. α-Tocopherol has also been shown to inhibit oxiapoptophagy (OXIdation + APOPTOsis + autoPHAGY), the complex mode of cell death induced not only by 7KC, but also by 7β-hydroxycholesterol and 24S-hydroxycholesterol [35,42]. In 158N murine oligodendrocytes, oxiapoptophagy is also inhibited by dimethyl fumarate (DMF), the active compound of Tecfidera/BG12 used in the treatment of the relapsing-remitting form of MS [43]. In human retinal pigment epithelial cells (ARPE-19), resveratrol (a polyphenol mainly found in red wine) showed cytoprotective effects and was able to inhibit VEGF-secretion [44]. Polyphenolic extracts of wine (epicatechin, epigallocatechin-3-gallate, caffeic acid) also prevent inflammation induced by a mixture of oxysterols representative of a hyper-cholesterolemic diet in CaCo-2 colonic epithelial cells [45,46]. Indicaxanthin, a bioactive pigment from cactus pear fruit, has also been shown to prevent 7KC-induced cell death in different cell types [47,48,49]. In 158N cells and SK-N-BE neuronal cells, the toxic effects of 7KC are also strongly inhibited by docosahexaenoic acid (DHA, C22:6 n-3), which is present at high levels in oily fish, such as sardines [35,36].

According to different studies, numerous compounds present in the Mediterranean diet (α-tocopherol, DHA, polyphenols) are able to impair the toxic effects of 7KC, and could help to prevent 7KC-associated diseases, including neurodegenerative diseases [28,50]. Thus, α-tocopherol, and γ-tocopherol, which are at elevated levels in olive oil (widely used in the Mediterranean diet), and which can cross the blood brain barrier [51,52], could be useful to attenuate the toxic effects of 7KC on microglial cells, which are thought to contribute to neurodegeneration. By analogy with DHA, which is a fatty acid, it is of interest to determine whether oleic acid (the major fatty acid of olive oil) is able to prevent 7KC-induced cellular dysfunction. Therefore, to evaluate the ability of major compounds present in the Mediterranean diet to prevent 7KC-induced microglial dysfunction, BV-2 murine microglial cells were cultured without or with 7KC associated or not with α-tocopherol, γ-tocopherol, Trolox (6-hydroxy-2,5,7,8-tetramethylchromane-2-carboxylic acid, a water soluble analog of Vitamin E), and oleic acid (OA; C18:1 n-9). Vitamin E is a fat-soluble vitamin which covers a set of eight organic molecules, four tocopherols (α-, β-, γ-, and δ-tocopherol) and four tocotrienols (α-, β-, γ-, and δ-tocotrienol). Some of the compounds studied (α-tocopherol, γ-tocopherol, OA), which are known to pass the blood brain barrier [51,52], are major components of olive oil, which is widely used in the Mediterranean diet. The ability of α-tocopherol, γ-tocopherol, Trolox, and OA to prevent the toxic effects of 7KC, especially their ability to impair 7KC-induced mitochondrial and peroxisomal dysfunction, was therefore evaluated in BV-2 cells.

## 2. Results and Discussion

### 2.1. Fatty Acid and Tocopherol Profile of Extra Virgin Olive Oils from Morocco, Spain and Tunisia

The performances of olive oils in terms of quality and composition depend on numerous parameters: genetic and environmental factors, especially climate and altitude but also the oil extraction process, soil type (arid or semi-arid, and irrigation), the temperature, rain, drought, fruit maturity and the harvest year [53,54,55,56]. It is thus important to determine the composition of extra virgin olive oils (EVOO) used as the reference in the present study and to establish whether particular compounds of these oils have cytoprotective effects on relevant in vitro models [57]. Usually, EVOO are characterized by high levels of oleic acid and are rich in tocopherols, which exhibit significant antioxidant activities [58,59]. Whatever the geographic origin of the EVOO studied (Morocco, Spain and Tunisia), the major fatty acid identified was oleic acid (OA; C18:1 n-9). Olive oil from Spain contained the largest quantity of OA (Spain > Morocco > Tunisia). High levels of the following fatty acids were also identified (descending order): C16:0 (palmitic acid), C18:2 n-6 (linoleic acid), C18:0 (stearic acid), C18:1 trans (vaccenic acid), C18:3 n-3 (α-linolenic acid) (Table 1). The different fatty acid profiles obtained by gas chromatography are shown in Figure S1. α-Tocopherol was the major tocopherol identified (Tunisia > Spain > Morocco) and γ-tocopherol was also detected (Spain > Tunisia > Morocco) (Table 1). Of note, previous in vivo experiments have shown that the Tunisian EVOO had protective effects against lipid peroxidation, measured on Wistar rat erythrocytes, liver and brain [60,61,62]. Data obtained on Tunisian EVOO indicate that it could have neuroprotective effects [62]. It was therefore of interest to determine the biological activities of major EVOO compounds (OA and tocopherols) on nerve cells.

These observations led us: (i) to determine the antioxidant properties of α-tocopherol, γ-tocopherol, Trolox, and OA with various conventional assays; and (ii) to evaluate the ability of these compounds to prevent the toxic effects of 7KC (mainly oxidative stress, and mitochondrial and peroxisomal dysfunction, which are major events known to be involved in neurodegeneration) on microglial cells, with the aim to ascertain the beneficial effects of major compounds (α- and γ-tocopherol, and OA) contained in the Mediterranean diet to prevent neurodegeneration.

### 2.2. Comparison of the Antioxidant Properties of α-Tocopherol, γ-Tocopherol, Trolox, and Oleic Acid with Three Complementary Techniques: The FRAP, DPPH and KRL Tests

The antioxidant properties of α-tocopherol, γ-tocopherol, Trolox, and OA were determined using three complementary methods: the Ferric Reducing Antioxidant Power (FRAP) assay, the 2,2-diphenyl-1-picrylhydrazyl (DPPH) radical scavenging assay and the KRL (Kit Radicaux Libres) test. With the different compounds studied (α-tocopherol, γ-tocopherol, Trolox and OA), these different techniques provided similar information (Table 2). When the antioxidant properties of α-tocopherol, γ-tocopherol, Trolox and OA were evaluated with the KRL test, they were determined at the following concentrations: 5, 10, 20, 50, and 100 μM. This test uses Trolox as the reference: one mol of α-tocopherol and γ-tocopherol have anti-oxidative properties equivalent to 0.94 and 1.31 mol of Trolox, respectively. With the KRL test, OA had no antioxidant properties but could modify the plasma membrane permeability of red blood cells used in the test; therefore no value was determined with the KRL test. With the FRAP and DPPH tests, the highest antioxidant capacity was observed with γ-tocopherol followed by α-tocopherol (Table 2). In agreement with the KRL test, OA showed no antioxidant potential with the FRAP test and the DPPH test (Table 2).

### 2.3. Evaluation of the Impact of α-Tocopherol, γ-Tocopherol, Trolox, and Oleic Acid on Mitochondrial Activity and/or Cell Growth with the MTT Test

**Figure 1:**
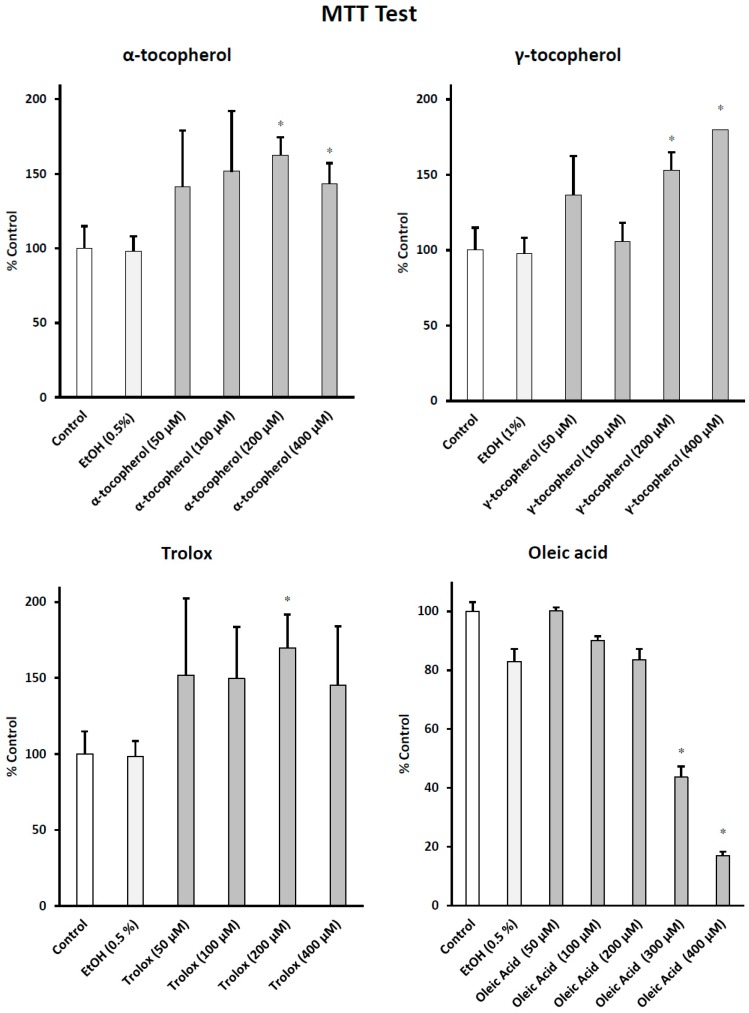
Effects of α-tocopherol, and γ-tocopherol, Trolox, and oleic acid on mitochondrial activity and/or cell growth. Murine microglial BV-2 cells were cultured for 24 h in the presence of α-, and γ-tocopherol, Trolox and oleic acid (OA) (50–400 μM) and the effect on mitochondrial activity and/or cell growth was determined with the MTT test. No difference was observed between control and vehicle (EtOH 0.5%–1%)-treated cells. The EtOH values correspond to the highest EtOH concentrations added to the culture medium: 0.5% with α-tocopherol, Trolox, and OA; and 1% with γ-tocopherol. Differences between vehicle and treated cells (* means significant differences by Mann–Whitney test; *p* ≤ 0.05). Data are mean ± SD of three independent experiments.

The impact of α-tocopherol, γ-tocopherol, Trolox, and OA, used at final concentrations of 50, 100, 200, and 400 μM, on mitochondrial activity and/or cell growth, was evaluated with the MTT test in BV-2 cells. The MTT showed no cytotoxic effects of α-tocopherol, γ-tocopherol, or Trolox whatever the concentration tested; MTT values were sometimes higher than in the control and in the vehicle (ethanol (EtOH))-treated cells (Figure 1). In addition, no significant differences were observed between control and vehicle-treated cells (Figure 1). OA at 300 and 400 μM inhibited mitochondrial activity and/or cell growth, but no cytotoxic effects were found at 50, 100 and 200 μM (Figure 1). Therefore, for further experiments, the compounds were used at the highest non-cytotoxic concentrations: 100–200 μM for OA; and 400 μM for α-tocopherol, γ-tocopherol, and Trolox.

### 2.4. Evaluation of the Effects of α-tocopherol, γ-Tocopherol, Trolox, and Oleic Acid on 7KC-Induced Loss of Mitochondrial Transmembrane Potential and Increased Permeability of Plasma Membrane and/or Cell Death Induction

**Figure 2:**
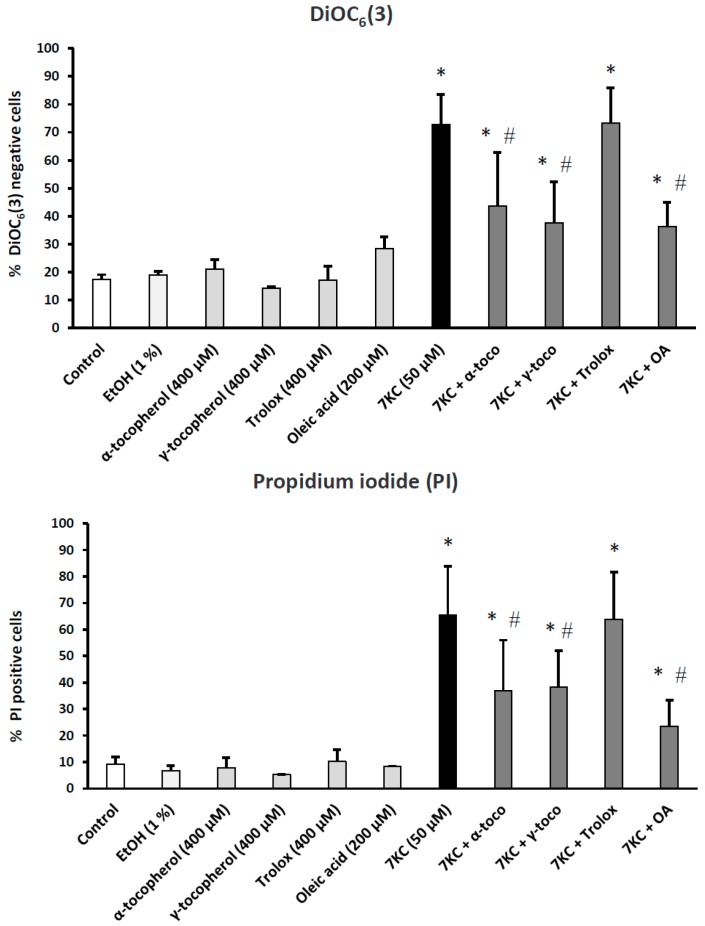
Protective effects of α-tocopherol, and γ-tocopherol, and oleic acid on 7-ketocholesterol-induced loss of mitochondrial transmembrane potential and plasma membrane damage (and/or cell death). Murine microglial BV-2 cells were cultured for 24 h in the presence of 7KC (50 μM) without and with α-, and γ-tocopherol (400 μM), Trolox (400 μM) and oleic acid (OA: 200 μM); the percentages of cells with depolarized mitochondria (characterized by a loss of mitochondrial transmembrane potential (ΔΨ_m_)) was determined by flow cytometry as was the percentage of propidium iodide-positive cells (dead cells and/or cells with damaged plasma membranes). No difference was observed between control and vehicle (EtOH 1%)-treated cells. The EtOH values correspond to the highest EtOH concentrations added to the culture medium: 1% with γ-tocopherol. Differences between vehicle and 7KC-treated cells (* means significant differences by Mann–Whitney test; *p* ≤ 0.05). Differences between 7KC-treated cells and (7KC + (α-tocopherol, γ-tocopherol, or oleic acid))-treated cells (# means significant differences by Mann–Whitney test; *p* ≤ 0.05). Data are mean ± SD of at least three independent experiments.

To determine the impact of α-tocopherol, γ-tocopherol, Trolox, and OA on 7KC-induced loss of mitochondrial transmembrane potential (ΔΨ_m_) and increased permeability of the plasma membrane (and/or cell death), BV-2 cells were stained with DiOC_6_(3) and propidium iodide (PI), respectively. Under these conditions, a marked decrease in ΔΨ_m_ (revealed by increased percentages of DiOC_6_(3) negative cells) and a marked increase in PI positive cells was observed with 7KC (Figure 2). In the presence of α-tocopherol, γ-tocopherol, and OA, these two major toxic effects of 7KC were strongly and significantly reduced (Figure 2). However, in the presence of Trolox, no protective effects were observed (Figure 2). It is noteworthy that the protective effects of α-tocopherol, γ-tocopherol, and OA evaluated with PI were similar when the compounds were added 2 h before 7KC or at the same time as 7KC (Figure S2).

### 2.5. Evaluation of the Effects of α-Tocopherol, γ-Tocopherol, Trolox, and Oleic Acid on 7KC-Induced Peroxisomal Dysfunction Determined by Abcd3 Protein Expression

**Figure 3:**
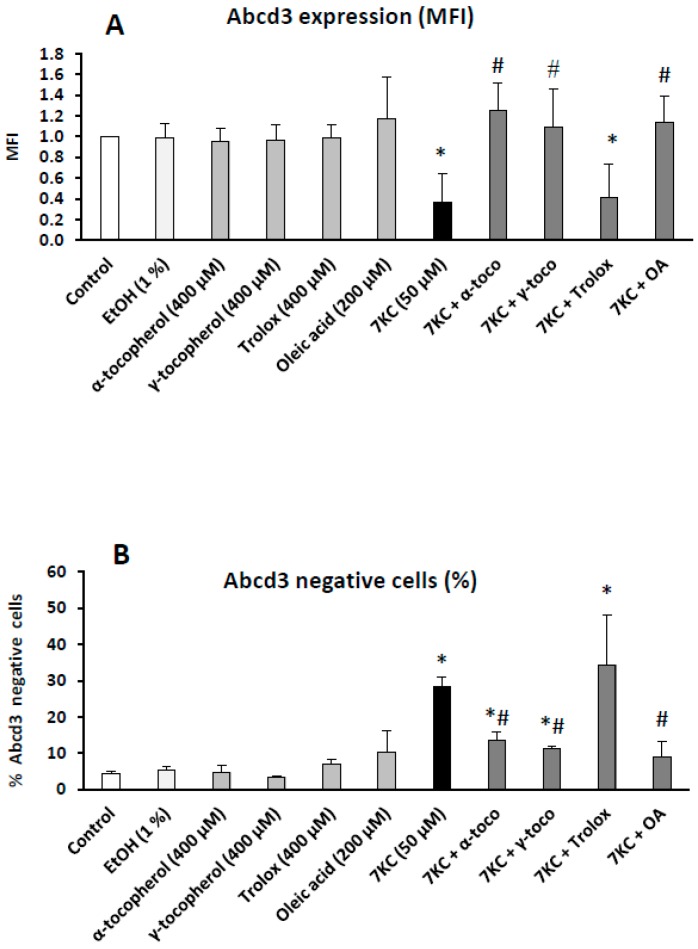
Protective effects of α-tocopherol, and γ-tocopherol, and oleic acid on 7-ketocholesterol-induced decreased expression of Abcd3, a marker of peroxisomal mass. Murine microglial BV-2 cells were cultured for 24 h in the presence of 7KC (50 μM) without and with α-, and γ-tocopherol (400 μM), Trolox (400 μM) and oleic acid (OA: 200 μM), and the expression of Abcd3 determined with the mean fluorescence intensity (MFI) was evaluated by flow cytometry (**A**); in addition, the percentage of Abcd3-negative cells was determined (**B**); No difference was observed between control and vehicle (EtOH 1%)-treated cells. The EtOH values correspond to the highest EtOH concentrations added to the culture medium: 1% with γ-tocopherol. Differences between vehicle and 7KC-treated cells (* means significant differences by Mann–Whitney test; *p* ≤ 0.05). Differences between 7KC-treated cells and (7KC + (α-tocopherol, γ-tocopherol, or oleic acid))-treated cells (# means significant differences by Mann–Whitney test; *p* ≤ 0.05). Data are mean ± SD of at least three independent experiments.

As the expression of the Abcd3 transporter is considered a suitable marker to evaluate peroxisomal mass, which can reflect peroxisomal biogenesis [63], the ability of 7KC to modulate the number of peroxisomes and/or peroxisomal mass was determined by flow cytometry with antibodies raised against Abcd3: the quantification of the mean fluorescence intensity (MFI) and the percentage of Abcd3 negative cells were determined (Figure 3). Under treatment with 7KC, the MFI of Abcd3-cells was decreased (Figure 3A) and the percentage of Abcd3-negative cells was increased (Figure 3B). By Western blotting, in agreement with data obtained by flow cytometric analysis, the decreased expression of Abcd3 was also observed [22]. These modifications of Abcd3 expression were significantly counteracted in the presence of α-tocopherol, γ-tocopherol, and OA, and values similar to those in control and vehicle (EtOH: 1%)-treated cells were observed (Figure 3). However, in the presence of Trolox, no protective effect was found (Figure 3).

### 2.6. Evaluation of the Effects of α-Tocopherol, γ-Tocopherol, Trolox, and Oleic Acid on 7KC-Induced Peroxisomal Dysfunction Evaluated by Abcd1, Abcd2, Abcd3, Acox1, and Mfp2 mRNA Levels

**Figure 4:**
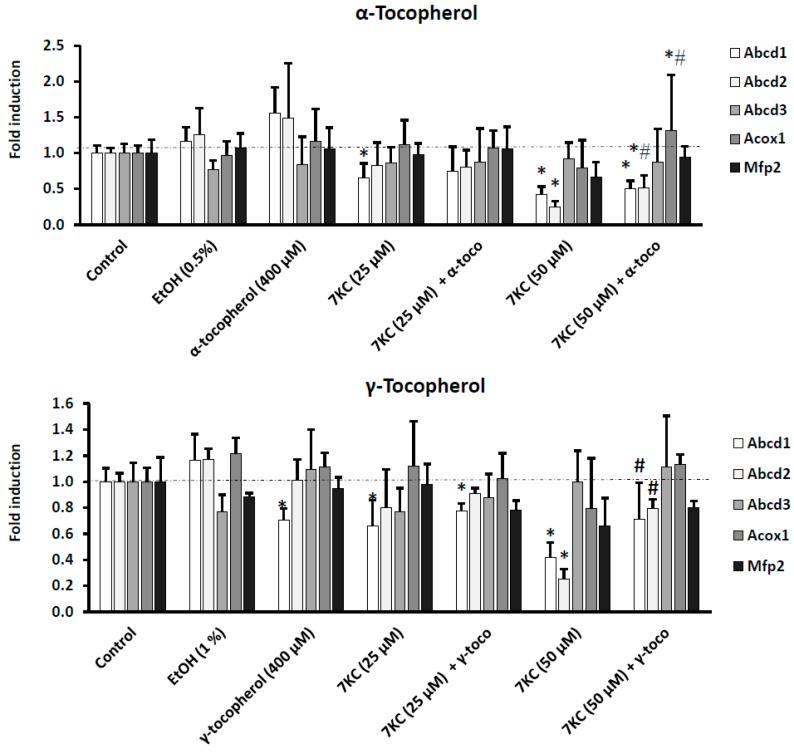

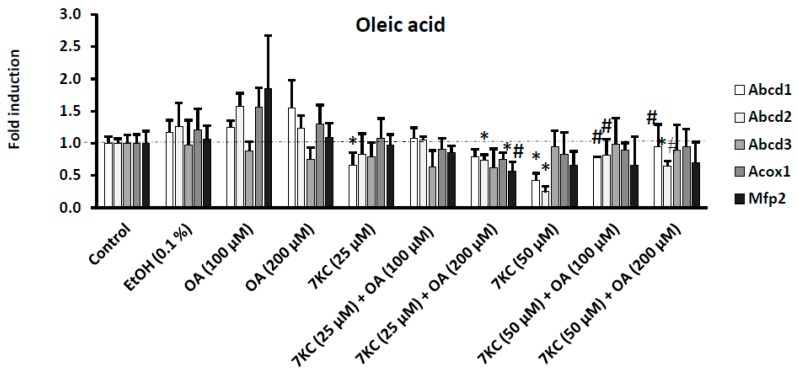
Effects of α-tocopherol, and γ-tocopherol, and oleic acid on 7-ketocholesterol-induced decreased transcription of Abcd1, Abcd2, Acox1 and Mfp2, specific proteins of peroxisomal β-oxidation, and on Abcd3 used as a marker of the peroxisomal mass. Murine microglial BV-2 cells were cultured for 24 h in the presence of 7KC (25–50 μM) without and with α-, and γ-tocopherol (400 μM), and oleic acid (OA: 100–200 μM). The mRNAs of the peroximal transporters (Abcd1, and Abcd2), and of the peroxisomal enzymes (Acox1, and Mfp2) as well as of Abcd3 (a marker of the peroxisomal mass) were quantified by RT-qPCR. To this end, 36B4 was used as the reference gene. No difference was observed between control and vehicle (EtOH 0.1%–1%)-treated cells. The EtOH values correspond to the highest EtOH concentrations added to the culture medium: 0.1% with OA, 0.5% with α-tocopherol, 1% with γ-tocopherol. Differences between control and 7KC-treated cells (* means significant differences by Mann–Whitney test; *p* ≤ 0.05). Differences between 7KC-treated cells and (7KC + (α-tocopherol, γ-tocopherol, or oleic acid))-treated cells (# means significant differences by Mann–Whitney test; *p* ≤ 0.05). Data are mean ± SD of at least three independent experiments.

**Figure 5:**
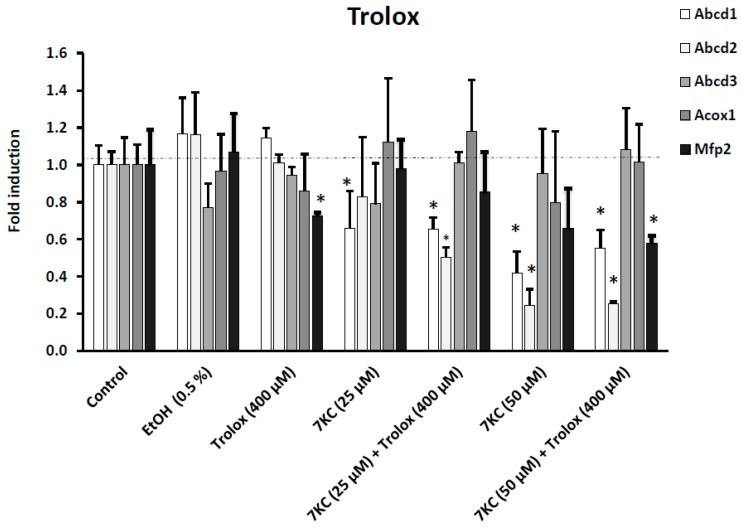
Effects of Trolox on 7-ketocholesterol-induced decreased transcription of Abcd1, Abcd2, Abcd3, Acox1 and Mfp2. Murine microglial BV-2 cells were cultured for 24 h in the presence of 7KC (25–50 μM) without and with Trolox (400 μM). The mRNAs of the peroxisomal transporters (Abcd1, and Abcd2), and of the peroxisomal enzymes (Acox1, and Mfp2) as well as of Abcd3 (a marker of the peroxisomal mass) were quantified by RT-qPCR. To this end, 36B4 was used as the reference gene. No difference was observed between control and vehicle (EtOH 0.1%–1%)-treated cells. The EtOH values correspond to the highest EtOH (0.5%) concentration added to the culture medium. Differences between control and 7KC-treated cells (* means significant differences by Mann–Whitney test; *p* ≤ 0.05). No differences between 7KC-treated cells and (7KC + (Trolox))-treated cells were observed. Data are mean ± SD of at least three independent experiments.

Abcd1 and Abcd2 are major peroxisomal proteins involved in the transport of VLCFA from the cytosol into peroxisomes for their breakdown by β-oxidation; Acox1 and Mfp2 are central oxidases of the β-oxidation pathway [64]. Abcd1, Acox1, and Mfp2 contribute to myelination and axonal integrity in the central nervous system, and their deficiency is associated with neurodegeneration [65]. It was therefore important to determine the effects of 7KC (25–50 μM) associated or not with α-tocopherol (400 μM), γ-tocopherol (400 μM), Trolox (400 μM) or OA (100–200 μM) on the corresponding mRNA levels of these proteins (Figure 4). The Ct values of the peroxisomal proteins associated with peroxisomal β-oxidation and of the reference genes were as follows: Abcd1: 26.8 ± 3.0; Abcd2: 24.6 ± 0.7; Acox1: 26.5 ± 2.1; Mfp2: 27.1 ± 0.8; and 36B4: 18.3 ± 1.2, and were in the range of those previously obtained [22]. It is noteworthy that the mRNAs levels of Abcd1 and Abcd2 were strongly affected in BV-2 cells treated with 7KC (25–50 μM), and a dose effect was observed; however, Acox1 was not affected and a significant decrease in the Mfp2 mRNA level was only observed under treatment with 7KC (50 μM); a slight effect of Trolox on the Mfp2 mRNA level was observed, and no effect of ethanol (used as vehicle) was found as compared with control cells (untreated cells) (Figure 4 and Figure 5). Interestingly, the decreased mRNA levels of Abcd1, Abcd2, and Mfp2 induced by treatment with 7KC (25–50 μM) was impaired in the presence of α-tocopherol, γ-tocopherol, and OA, but remained lower than those in control and vehicle-treated cells (Figure 4). As expected, Trolox did not restore the marked decrease in Abcd1 and Abcd2 mRNAs levels induced by 7KC (50 μM), or the slight decrease in the Mfp2 mRNA level induced by 7KC (50 μM) (Figure 5). In addition, as Abcd3 protein, considered a suitable marker of peroxisomal mass [63], was decreased as determined by flow cytometry and Western blotting, its mRNA level was also quantified (Ct value of Abcd3 in untreated BV-2 cells = 26.5 ± 0.7): under treatment with 7KC, no significant modification of the Abcd3 mRNA level was observed, and no effect of α-tocopherol, γ-tocopherol, Trolox and OA on the Abcd3 mRNA level was found (Figure 4).

### 2.7. Discussion

In numerous degenerative diseases, increased oxidative stress and mitochondrial dysfunction are observed. These events are therefore thought to play important roles in the pathophysiology of neurodegeneration, and a major effort is being made to identify molecules able to prevent mitochondrial dysfunction and oxidative stress in these diseases. However, few data concerning the role played by peroxisome dysfunction in these diseases are available even though the role of this organelle in demyelination [66,67] and in the control of the RedOx status [32,68] is well documented. In addition, there is substantial evidence that peroxisomal deficiencies and/or dysfunction are able to severely alter the morphology and metabolism of mitochondria [69,70]. Therefore, peroxisomes could constitute a new and potential target for innovative therapies in the treatment of neurodegeneration. It is therefore of interest to identify molecules able to prevent not only mitochondrial dysfunction and oxidative stress but also peroxisomal dysfunction. We report here that major compounds of the Mediterranean diet are able to prevent 7KC-induced mitochondrial and peroxisomal dysfunction in murine microglial BV-2 cells. These results are of interest given the pro-oxidative environment induced by 7KC, levels of which are increased in the cerebrospinal fluid of patients with MS [21], in the plasma of patients with X-ALD [22] and in those with Niemann–Pick disease [23], in the arteries of patients with atherosclerosis [27] and in the retina of patients with age related macular degeneration [71,72].

Currently, impaired mitochondrial respiration and function are proposed as the main causes of several neurodegenerative diseases, including prototypic diseases of the central nervous system white matter such as MS and X-ALD [73,74,75], which are associated with increased levels of 7KC in the plasma and/or cerebrospinal fluid of patients [21,22]. In MS, mitochondria play central roles in axonal neurodegeneration [76]. Since mitochondrial dysfunction plays key roles in 7KC-induced cell death, it is important to determine the precise effect of 7KC at the mitochondrial level and to identify molecules able to impair the deleterious effects of 7KC on mitochondria [27]. As previously shown in 158N murine oligodendrocytes and in murine microglial BV-2 cells, 7KC-induced cell death is associated with mitochondrial dysfunction [30] leading to a loss of mitochondrial transmembrane potential (ΔΨ_m_), which is reversed by α-tocopherol [34,35,42]. Whereas no protective effects were observed with γ-tocopherol in A7R5 rat aorta smooth muscle cells [41], 7KC-induced loss of ΔΨ_m_ and increased permeability to PI (which is a criterion of cell death) were counteracted by γ-tocopherol in BV-2 cells. However, in the presence of Trolox, which is a water-soluble analog of α-/γ-tocopherol with potent antioxidant properties determined with the FRAP, DPPH and KRL tests, no protective effects were observed. These data support the hypothesis that the protective effect of α-tocopherol does not depend on the cell type considered, whereas the protective effect of γ-tocopherol is cell-type specific. As Trolox, which is a strong antioxidant, provided no protection against the toxic effects of 7KC, our data suggest that the anti-oxidative properties of α- and γ-tocopherol are not essential to prevent these toxic effects of 7KC, and that the protective activities of these tocopherols involve other mechanisms [38,39,40,77]. Our observations are in agreement with data reporting that all of the stereoisomers and derivatives of Vitamin E have antioxidant activities and that small differences exist for each form [38,39,78]. Therefore, to explain the differences between α-/γ-tocopherol and Trolox, the biological properties of these molecules must be taken into consideration. Importantly, α-tocopherol has been shown to decrease the accumulation of 7KC in lipid rafts on A7R5 and 158N cells [34,41]. It has been suggested that the ability of α-tocopherol to impair 7KC-induced cell death (both in A7R5 and 158N cells) depends not only on its anti-oxidative properties, but also its ability to impair 7KC-accumulation in lipid raft microdomains, which triggers GSK-3 activation, thus leading to mitochondrial dysfunction and apoptosis [34,41,79]. In the experiments of Royer et al. [41], α- and γ-tocopherol were used at 100 μM. However, in our experiments, they were used at 400 μM. It is suggested, that at elevated concentrations, which could be reached over a short period of time in vivo, similar properties of α- and γ-tocopherol (impaired 7KC-accumulation in lipid rafts) could be obtained. Our data also underline that α-tocopherol is more effective than γ-tocopherol to prevent the toxic effects of 7KC, including loss of ΔΨ_m_ and increased permeability to PI, which is associated with membrane damage and/or cell-death induction [80,81]. Whereas the absence of protective effects with Trolox can be surprising since it is a potent antioxidant molecule, the ability of this compound to prevent side effects associated with oxidative stress (mainly cell death induction) seems to be extremely variable. Thus, on human monocytic U937 cells, Trolox prevents 7β-hydroxycholesterol-induced apoptosis but does not protect against cell death induced by β-epoxide [82]. Therefore, the protective effect of Trolox for a given cell type can depend on the oxysterol considered. On human myocytes, it has also been reported that Trolox is effective against the hypoxanthine-xanthine oxidase free radical generation system whereas it has no effects on human fibroblasts and human endothelial cells [83]. To explain these differences, some hypotheses can be made: (i) The antioxidant activity of Trolox could be radical specific; indeed, Trolox has been described as a peroxyl-radical (ROO^•^) scavenger; (ii) Trolox could also act in synergy with other antioxidants; thus, depending on the cytotoxic molecule used, the more or less important depletion of antioxidants in the plasma membrane may influence the activity of Trolox [38,39,84]. As inflammatory processes play central roles in neurodegeneration, and as 7KC is also a potent inducer of inflammation [27], it could be interesting, by analogy with data obtained with lipopolysaccharide (LPS) [85], to determine the impact of mitochondrial dysfunction on the phenotype of BV-2 cells (M1: pro-inflammatory; M2: anti-inflammatory), and the ability of α- and γ-tocopherol and OA to modulate the phenotype. As the olive oils tested showed high α-tocopherol/γ-tocopherol ratios (from 3.5 to 12.5), our data support the potential benefit of olive oil consumption to prevent the toxic effects of 7KC associated with numerous age-related diseases including neurodegenerative diseases [28]. In addition, even though OA had no anti-oxidative properties, it was as effective as α- and γ-tocopherol in preventing both 7KC-induced loss of ΔΨ_m_ and increased permeability to PI in microglial BV-2 cells. These data show that two classes of molecules can afford protection against 7KC-induced cytotoxicity: those with antioxidant properties such as α-tocopherol and γ-tocopherol; those with no antioxidant properties such as fatty acids (OA, DHA) [35] and dimethyl fumarate (DMF), the active compound of Tecfidera/BG12 used in the treatment of the relapsing-remitting form of MS [43]. This demonstrates that molecules able to prevent the toxic effects of 7KC can be active by involving mechanisms other than those associated with their antioxidant power. With tocopherols, several mechanisms are also probably involved: (i) impairment of 7KC accumulation in lipid rafts [34,41]; (ii) changes in the surface exposure of membrane receptors; and (iii) modulation of signal transduction by modifying plasma membrane properties [77,86]. Moreover, as it has been reported that the cytoprotective properties of α-tocopherol are mostly related to gene regulation rather than to antioxidant activity in toxin-induced cell death in hepatocytes [87], similar mechanisms cannot be excluded in the ability of tocopherols to prevent the toxic effects of 7KC. Indeed, several studies have reported that numerous genes involved in major cellular functions (anti-oxidant defense, inflammation and cell adhesion, lipid homeostasis, etc.) affected by 7KC are regulated by Vitamin E [38,39]. With OA, it has been hypothesized that the integration of this fatty acid into cardiolipin would generate non-oxidizable cardiolipin species able to protect cells against apoptosis [88]. It has also been reported that OA protects INS-1E β-cells from palmitate-induced apoptosis by suppressing endoplasmic reticulum stress and that this effect was independent of chaperone activation [89]. More recently, nutrigenomics data on the functional components of olive oil such as OA, biophenols and vitamin E, point towards gene regulation activities of these components [90]. In addition, several components of extra virgin olive oil (EVOO), including OA have been shown to prevent genomic instability, telomere attrition, epigenetic alterations, mitochondrial dysfunction, cellular senescence, and altered intracellular communications, all of which are hallmarks of aging and could be under the control of numerous oxysterols including 7KC [28,91]. Interestingly, the fact that no differences in the cytoprotective activities of OA, α- and γ-tocopherol were observed between simultaneous and pre-treatment with 7KC reveals the immediate cytoprotective effect of these molecules. This observation has important therapeutic implications. Indeed, these compounds (OA, and α- and γ-tocopherol), when supplied by an appropriate diet (such as the Mediterranean diet) or when included in functional foods, could be used to prevent neurodegeneration. These data also support the notion that OA, α- and γ-tocopherol, which have the ability to cross the blood brain barrier, could also be used for the treatment of neurodegenerative diseases or other age-related diseases. Altogether, our data establish that among the major compounds present in olive oils, OA, and α- and γ-tocopherol have strong cytoprotective properties able to act against the toxic effects of 7KC associated with mitochondrial dysfunction.

At the moment, the role played by mitochondria in different types of cell death is well documented, as is the involvement of other organelles such as lysosomes and the endoplasmic reticulum [92,93]. However, little is known about the impact of cytotoxic compounds on peroxisome biogenesis and activity, or about the contribution of peroxisomes to cell death [33]. As 7KC could contribute to the pathophysiology of MS and X-ALD [21,22], we wondered whether it could have various side effects on the peroxisome, which is essential for RedOx homeostasis and the synthesis of myelin by oligodendrocytes [66,94] and whose deficiency in nerve cells of the brain produces astrogliosis and gliosis associated with inflammatory processes in the central nervous system [67]. As deficient peroxisomal β-oxidation fosters the accumulation of very long chain fatty acids (VLCFA) in X-ALD patients, and might also occur in patients with dementia and MS [95,96,97], we determined by RT-qPCR and/or flow cytometry the impact of 7KC on mRNA and/or protein levels of Abcd1, Abcd2, Mfp2 and Acox1, which are involved in the peroxisomal β-oxidation pathway of VLCFA [64,98] and of Abcd3 used as a marker of peroxisomal mass [63]. In agreement with our previous data, which also included protein analysis of Abcd1, Abcd2, and Abcd3 by Western blot as well as the measurement of catalase and Acox1 activities [22], we found marked decreases in Abcd1, Abcd2, Mfp2 mRNAs levels, suggesting impaired β-oxidation in 7KC-treated BV-2 cells. We also observed an increased percentage of cells with reduced levels of Abcd3, suggesting impaired peroxisomal biogenesis, which may contribute to neuronal dysfunction and degeneration in MS [63]. As defects in proteins (transporters and enzymes) involved in the peroxisomal β-oxidation pathway can increase oxidative stress (overproduction of superoxide anions, hydrogen peroxide and nitrous oxide) [94], which can in turn initiate and/or amplify mitochondrial dysfunction [32,68], it is important to identify molecules able to prevent peroxisomal dysfunction, especially in a pro-oxidative environment, which is a hallmark of age-related and neurodegenerative diseases. As there are close functional and metabolic connections between the mitochondria and peroxisomes [99], and as numerous products present in the Mediterranean diet, such as α-tocopherol, DHA, and polyphenols, are known to prevent 7KC-induced mitochondrial dysfunction [35,42,44,48,49], it was also important to determine the impact of some of these compounds on 7KC-induced peroxisomal dysfunction. Importantly, as OA has been reported to favor peroxisomal biogenesis in yeast [100], our data support the notion that it could be of interest to determine its effects on human peroxisomes. Interestingly, and for the first time, we established that α- and γ-tocopherol and OA but not Trolox exerted protective effects against 7KC-induced peroxisomal dysfunction in microglial cells.

The cell targets and the signaling pathways involved in the protective effects of these molecules now have to be determined. It is necessary to establish whether the protective mechanisms of α-tocopherol at the mitochondrial level in BV-2 cells are similar to those observed in 158N cells (recovery of ATP production, restoration of oxidative phosphorylation and tricarboxilic acid cycle activity) [30], and whether or not the protective effects of γ-tocopherol and OA at the mitochondrial level are similar to those of α-tocopherol. If we consider that ATP production at the mitochondrial level is necessary for peroxisomal β-oxidation, especially for the import of VLCFA into peroxisomes via ABCD 1 and 2 transporters, it can be supposed that the recovery of peroxisomal β-oxidation depends on the recovery of mitochondrial activity. As peroxisomal β-oxidation is involved in the control of the RedOx status, and of ROS and RNS production [94], considered the first hit leading to inflammation, demyelination and/or neurodegeneration [2,3], the consumption of molecules such as α- and γ-tocopherol and OA could help to prevent the onset and progression of various neurodegenerative diseases. It has been reported that a high-quality diet assessed by comparison with the Mediterranean diet may decrease the risk of MS [101]. The difference in dietary habits between Nordic and Mediterranean countries may also explain, at least in part, why the incidence of MS is higher in Nordic countries. Moreover, there is evidence that some dietary compounds, especially fatty acids, can influence brain inflammation and play key roles in neurodegeneration via connections between the small intestine and the brain [102]. Altogether, these arguments support the notion that the consumption of α- and γ-tocopherol and OA may help to prevent different forms of neurodegeneration [103].

**Figure 6:**
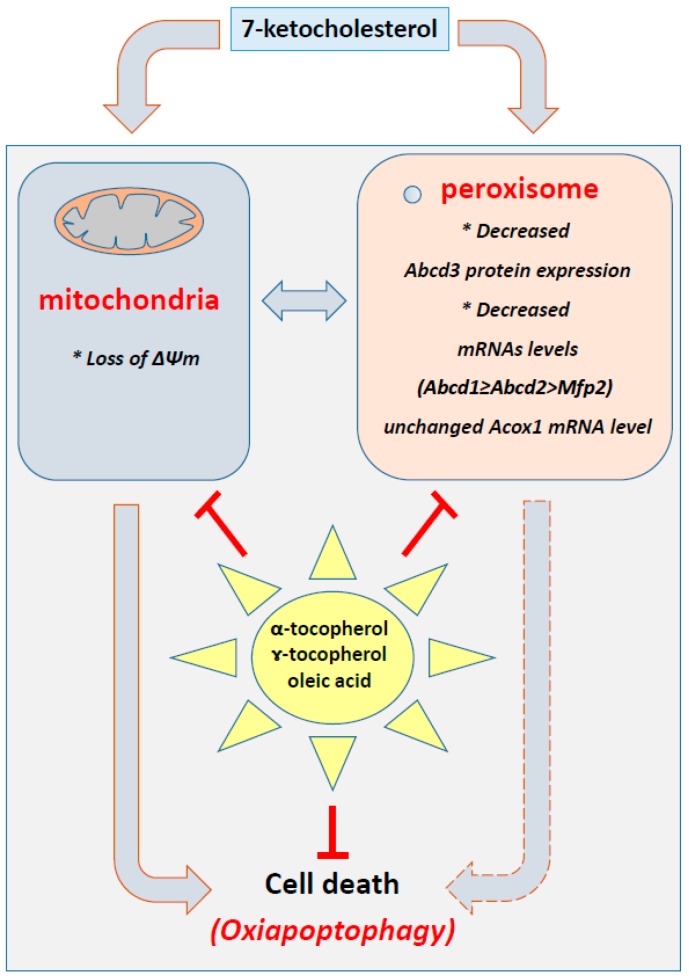
Impairment of 7-ketocholesterol-induced mitochondrial and peroxisomal dysfunction with α-tocopherol, and γ-tocopherol, and oleic acid, major compounds of the Mediterranean diet, in murine microglial BV-2 cells. Under treatment with 7-ketocholesterol, which is found at increased levels in the plasma and cerebrospinal fluid of patients with demyelinating diseases, such as multiple sclerosis (MS) and X-ALD [21,22], and which is found at elevated concentrations in the retina and the arterial wall of patients with age related macular degeneration and atherosclerosis, respectively [27,71,72], α-, and γ-tocopherol, and oleic acid (but not Trolox) were able to prevent major mitochondrial and peroxisomal dysfunctions: loss of mitochondrial transmembrane potential (ΔΨ_m_), which is known to trigger cell death defined as oxiapoptophagy (OXIdation + APOPTOsis + autoPHAGY) [35,42]; decreased expression of Abcd3, considered a marker of mitochondrial mass, and decreased transcription of Abcd1 and Abcd2, two specific transporters involved in peroxisomal β-oxidation, whose inactivation favors the accumulation of very long chain fatty acids, which are known to trigger oxidative stress. These data support the hypothesis that major compounds of the Mediterranean diet, and consequently eating habits, may have important impacts on the biogenesis and activity of major organelles, such as mitochondria and peroxisomes, which are closely connected and known to play major roles in neurodegeneration.

In conclusion, the present study brings new information on organelle dysfunction associated with 7KC-induced cell death: 7KC not only induces mitochondrial and lysosomal alterations [35,104], and endoplasmic reticulum stress [105], but also peroxisomal changes that underlie alterations in peroxisomal biogenesis (decreased expression of Abcd3) and functions (decreased mRNAs levels of Abcd1, and Abcd2, especially). In addition, our data show that tocopherols (α- and γ-tocopherol) and OA, but not Trolox, are able to prevent 7KC-induced mitochondrial/peroxisomal dysfunction and cell death in murine microglial BV-2 cells (Figure 6). It is therefore suggested that these molecules, which are present in the Mediterranean diet, and constitute major compounds of olive oil, might help to prevent major neurodegenerative diseases associated with mitochondrial and/or peroxisomal alterations, which contribute to microglial dysfunction.

## 3. Materials and Methods

### 3.1. Cell Culture and Treatments

Murine microglial cells (BV-2) were from Banca-Biologica Cell Factory (IST Genoa, Italy). They were seeded at 1.2 × 10^6^ cells in tissue culture dishes (100 × 20 mm, FALCON, Corning, Tewksbury, MA, USA) (with 10 mL of culture medium), or at 60 × 10^3^ cells per six-well plate (with 2 mL of culture medium). They were cultured in RPMI 1640 (Lonza, Amboise, France) supplemented with 10% (*v*/*v*) heat-inactivated fetal calf serum (FCS) (Dutscher, Brumath, France) and 1% antibiotics (penicillin, streptomycin) (Dutscher). The cells were incubated at 37 °C in a humidified atmosphere containing 5% CO_2_. For subcultures, cells were detached by pipetting, and passaged twice a week.

7KC (Ref: C2394) was from Sigma-Aldrich (St. Quentin Fallavier, France). The stock solution of 7KC was prepared at 800 μg/mL (2 mM) as previously described [34]. The stock solutions of oleic acid (OA; Sigma-Aldrich) were prepared as follows: 20 mg/mL (70 mM) in 10% ethanol. For cell treatment, OA was used at various concentrations (50, 100, 200, and 400 μM), and the final corresponding ethanol concentrations were: 0.06%, 0.03%, 0.015%, and 0.0075%, respectively. 7KC concentrations (10 and 20 μg/mL (corresponding to 25 and 50 μM)) and the time of treatment (24 h) were chosen because they are known to induce mitochondrial and peroxisomal dysfunction [22,30,79].

After 24 h of culture, BV-2 cells were incubated with 7KC (25 and 50 μM) for 24 h. When the cells were treated with 7KC associated with α-tocopherol (Sigma-Aldrich), γ-tocopherol (Sigma-Aldrich), Trolox (Sigma-Aldrich), or OA (50, 100, 200 and/or 400 μM), these compounds were added to the culture medium 2 h before the 7KC.

The concentrations used for 7KC, tocopherols and OA were chosen for the following reasons. In vitro, the cytotoxic effect of 7KC is evaluated by its 50% inhibiting concentration (IC50) value, which is in the range of 25–50 μM in different types of cells [28]. These IC50 values are 500 to 1000 times higher than in the plasma of aged subjects or hypercholesterolemic patients [28]. However, it is important to consider that oxysterol concentrations normalized to cholesterol were about 43 times higher in carotid plaque than in plasma [106]. Moreover, when 7KC is used at concentrations in the range of IC50 values, which are concentrations commonly used in toxicological studies in order to permit comparisons from one compound to another, only 10%–35% accumulate within the cell [107,108]. Interestingly, similar observations were made with 27-hydroxycholesterol [109]. Based on these considerations, it can be supposed that the intracellular oxysterol content obtained in vitro could be in the range of order of those occurring in vivo, even though it must also be taken into account that important variations can occur inside a tissue from one cell type to another. Furthermore, it is important to emphasize that the use of 7KC (25–50 μM) on different cell types is a relevant model: (i) to evaluate the relationship between oxidative stress, apoptosis and autophagy; (ii) to specify the part played by mitochondria and peroxisomes in these processes; and (iii) to determine the interactions between these two organelles in lipotoxicity [22,32,68]. OA and tocopherols were used at the highest non-cytotoxic concentrations able to prevent 7KC-induced apoptosis [34]. With α-tocopherol, it has been previously reported that the strongest cytoprotective effects on 158N cells were observed at 200 and 400 μM [34]. In the serum, the concentration of OA in normal subjects is in the range of 500 μM [110]. Therefore, in the present study, OA was used at 200 μM, and α- and γ-tocopherol at 400 μM.

### 3.2. Determination of the Fatty Acid Profile of Olive Oils by Gas Chromatography

The olive oils from Morocco and Tunisia were Extra Virgin Olive Oils (EVOO), obtained from artisanal manufactures and were simply made by crushing olives and extracting the juice. They were stored at 4 °C until analysis, and were analyzed between 6 and 12 months after being obtained. The Spanish olive oil was also an EVOO, and is commercially available. Lipids were extracted from the different oils (Morocco, Spain, Tunisia (Mahdia)) according to the Moilanen and Nikkari method [111]. C19:0 was used as the internal standard (IS). Lipids were transmethylated using boron trifluoride in methanol according to Morrison and Smith [112]. Fatty acid methyl esters were subsequently extracted with hexane and analyzed using gas chromatography on a Hewlett Packard Model 5890 gas chromatograph (Palo Alto, Santa Clara, CA, USA) using a CPSIL-88 column (100 m × 0.25 mm i.d., film thickness 0.20 μm; Varian, Les Ulis, France) equipped with a flame ionization detector (FID). Hydrogen was used as the carrier gas (inlet pressure, 210 kPa). The oven temperature was held at 60 °C for 5 min, increased to 165 °C at 15 °C/min and held for 1 min, and then to 225 °C at 2 °C/min and finally held at 250 °C for 17 min. The injector and the detector were maintained at 250 °C and the samples were injected. Fatty acid methyl esters were identified by comparison with commercial and synthetic standards (Sigma Aldrich, St. Quentin-Fallavier, France). The data were processed using EZChrom Elite software (Agilent Technologies, Massy, France) and reported as mg/g of total lipids.

### 3.3. Determination of the Tocopherol Profile of Olive Oils by High Pressure Liquid Chromatography

Forty milligrams of oil (Morocco, Spain, Tunisia (Mahdia)) was resuspended in 1 mL of a mixed high performance liquid chromatography (HPLC) mobile phase: acetonitrile/methanol containing 50 mM ammonium acetate/water/dichloromethane (700:150:50:100, *v*/*v*/*v*/*v*). After resuspension, the extract was vortexed for 30 s. Samples of 80 μL were injected into the HPLC system for the analysis of Tocopherols. The analytical conditions were based on those reported by Lyan et al. [113]. The HPLC apparatus was a Jasco PU-1580 Plus intelligent pump equipped with an automatic injector system AS300 (Thermo Finnigan, les Ulis, France) and a Jasco MD-1510 plus multi-wavelength detector (JASCO International Co., Ltd., Lisses, France). HPLC analyses were carried out using RPHPLC with a Nucleosil C18 column (250 × 4.6 mm i.d., 5 μm particle size) and a VIDAK C18 column (250 × 4.6 mm i.d., 5 μm particle size) under isocratic conditions. The mobile phase consisted of a mixture of acetonitrile/methanol at 50 mM ammonium acetate/water/dichloromethane (700:150:50:100, *v*/*v*/*v*/*v*), at a flow rate of 2 mL/min. The tocopherols were detected at 298 nm, their identification was ensured by comparing the retention times and absorption spectra with reference standards and their quantification was ensured using standard curves for each compound. Six quantities of α-tocopherol (range: 0.5–8.5 μg), γ-tocopherol (range: 1–25 μg) and δ-tocopherol (range: 0.1–2 μg) were injected into the HPLC system (each standard being dissolved in 1 mL of the HPLC mobile phase: acetonitrile/methanol containing 50 mM ammonium acetate/water/dichloromethane (700:150:50:100, *v*/*v*/*v*/*v*); the linear regression equation for each standard curve was then obtained by plotting the amount of the standard compound injected against the peak surface area. The regression equation and correlation coefficient (r2) were calculated using ChromNav software (JASCO International Co.).

### 3.4. Ferric Reducing Antioxidant Power Assay

The ferric reducing antioxidant power (FRAP) assay measures the antioxidant potential of compounds or of mixtures of compounds through the reduction of ferric iron (Fe^3+^) to ferrous iron (Fe^2+^) [114,115]. This method was used to compare the antioxidant potential of α-tocopherol, γ-tocopherol, and OA as compared with Trolox used as the positive reference. Briefly, α-tocopherol, γ-tocopherol, OA and Trolox were prepared in a range of concentrations from 0 to 6 mg/mL in phosphate buffer (PB: 0.2 M, pH 6.6) and potassium ferrocyanide (PF: 1% *w*/*v*; Fluka) mixed in equal volumes (PB (200 μL) + PF (200 μL)). The mixture was incubated at 50 °C for 20 min. Then, 200 μL of trichloroacetic acid (TCA: 10% *w*/*v*) (Sigma) was added to the reaction mixture. After centrifugation (1000× *g*, 10 min), 500 μL deionized water and ferric chloride (0.5 mL; 0.1% *w*/*v*) (Carlo-Erba, Val-de-Reuil, France) were added. After 30 min, the absorbance was measured at 700 nm (Sunrise spectrophotometer, Tecan, Lyon, France). The antioxidant potential of α-tocopherol, γ-tocopherol, and OA evaluated with the FRAP method was estimated in Trolox equivalent (1 mole of α-tocopherol, γ-tocopherol, and OA is equivalent to X mole of Trolox; X was determined with the calibration curve obtained with Trolox).

### 3.5. DPPH Assay

The DPPH assay uses the 2,2-diphenyl-1-picrylhydrazyl (C_18_H_12_N_5_O_6_) (Sigma Aldrich), which can be regarded as a stable free radical [116]. Scavenging of DPPH free radical is the basis of a common antioxidant assay which is frequently used to measure the anti-radical activity of compounds or of mixtures of compounds. The free purple form of DPPH (diphenyl-picryl hydrayl) is reduced by antioxidants to DPPH (2,2-diphenyl-picryl hydrazine), which is yellow [117]. The solution of DPPH was dissolved in absolute methanol (0.2 mM). One hundred microliters of the sample to be tested was added to 100 μL of the DPPH solution. After stirring the mixture with a vortex, the tubes were placed in the dark at room temperature for 30 min. The absorbance was read at 517 nm (Sunrise spectrophotometer, Tecan, Lyon, France). The negative control contained 100 μL of water and 100 μL of the DPPH solution. The antioxidant potential of α-tocopherol, γ-tocopherol, and OA evaluated with the DPPH method was estimated in Trolox equivalents (1 mole of α-tocopherol, γ-tocopherol, and OA is equivalent to X mole of Trolox; X was determined with the calibration curve obtained with Trolox).

### 3.6. KRL Test

The antioxidant potential of α-tocopherol, γ-tocopherol, and oleic acid (OA) was evaluated with the KRL (Kit Radicaux Libres) test [35,118]. Briefly, the oxidation of red blood cells by molecular oxygen was performed in an aqueous suspension using the azo-compound 2-2′-azo-bis-(2-amidinopropane) hydrochloride (AAPH) as the free radical initiator without or with α-tocopherol, γ-tocopherol, Trolox, and OA used at (5, 10, 20, 50 and 100 μM). Several parameters were calculated from the time-dependent curve of AAPH-induced hemolysis. The time required to achieve 50% hemolysis measured by the optical density of hemoglobin was determined (red blood cell half-hemolysis time in min) as was the equivalent of mmol Trolox/mol of α-tocopherol (γ-tocopherol, or OA) and the equivalent of mmol of Gallic acid/mole of α-tocopherol (or γ-tocopherol, or OA), which permits a comparison between the two molecules independently of their concentrations. Hemolysis was recorded using a 96-well microplate reader by measuring the optical density decay at 450 nm (Sunrise spectrophotometer, Tecan, France). For each well, absorbance measurements were performed 75 times, once every 150 s.

### 3.7. Colorimetric MTT Assay

The MTT assay was used to evaluate the effects of treatments on cell proliferation and/or mitochondrial activity. The MTT assay was carried out on BV-2 cells plated in 6-well flat-bottom culture plates after 24 h of treatment withα-tocopherol, γ-tocopherol, Trolox and OA (50, 100, 200, and 400 μM). MTT salt is reduced to formazan in the metabolically active cells by the mitochondrial enzyme succinate dehydrogenase to form NADH and NADPH [119]. The plates were read at 570 nm with a microplate reader (Sunrise, Tecan, France).

### 3.8. Measurement of Mitochondrial Transmembrane Potential with DiOC_6_(3)

Variations in the mitochondrial transmembrane potential were measured with 3,3′-dihexyloxacarbocyanine iodide (DiOC_6_(3)) (Life Technologies, Gaithersburg, MD, USA). Cells were stained with DiOC_6_(3) (40 nM) as previously described [22]. Mitochondrial depolarization is indicated by decreased green fluorescence collected through a 520/20 nm band pass filter on a Galaxy flow cytometer (Dako/Partec, Münster, Germany). Ten thousand cells were acquired; data were analyzed with Flomax (Partec, Münster, Germany) or FlowJo (Tree Star Inc., Ashland, OR, USA) software.

### 3.9. Measurement of Plasma Membrane Permeability and/or Cell Death with Propidium Iodide

BV-2 cells were stained with 1 μg/mL of propidium iodide (PI), which enters dead cells or cells with damaged plasma membranes only [81]. Fluorescence was collected using a 590/10 nm bandpass filter. Flow cytometric analyses were performed on a Galaxy flow cytometer (Dako/Partec). Ten thousand cells were acquired for each sample. Data were analyzed with Flomax software (Partec).

### 3.10. Flow Cytometric Quantification of Abcd3 Expression

Cells were detached by pipetting, and fixed with paraformaldehyde (Sigma-Aldrich) diluted in PBS (2% *v*/*v*, room temperature, 15 min). After two washes in PBS, the cells were incubated at room temperature for 30 min in PBS/10% (*v*/*v*) FCS (Dutscher)/0.05% (*w*/*v*) saponin (Sigma-Aldrich). After washing in PBS, the cells were incubated with a rabbit polyclonal antibody raised against Abcd3 (ref: #11523651, Pierce/Thermo Fisher Scientific, Brumath, France). The cells were incubated for 1 h at room temperature with the Abcd3 antibody diluted (1/500) in PBS/10% (*v*/*v*) FCS/0.05% (*w*/*v*) saponin (Sigma-Aldrich). After 2 washes in PBS, the cells were incubated for 30 min in the dark with a goat anti-rabbit secondary antibody coupled with Alexa 488 (Santa-Cruz Biotechnology, Santa Cruz, CA, USA) (1/500) in PBS/10% (*v*/*v*) FCS/saponin 0.05% (*w*/*v*). After washing in PBS, the cells were resuspended in PBS and immediately analyzed by flow cytometry on a Galaxy flow cytometer (Dako/Partec). Fluorescence was collected through a 520/20 nm bandpass filter. For each sample, fluorescence was quantified on 10,000 cells, and data were analyzed with FlowJo software (Tree Star Inc., Ashland, OR, USA). Absolute and conjugated controls (cells without antibodies, and without primary antibody, respectively) were realized for the different conditions of treatments.

### 3.11. Quantification of Abcd1, Abcd2, Abcd3, Acox1, and Mfp2 mRNAs by RT-qPCR

Total mRNA from BV-2 cells was extracted and purified using the RNeasy Mini Kit (Qiagen, Hilden, Germany) with 15 min DNAse treatment (Qiagen). Total mRNA concentration was measured with TrayCell (Hellma, Paris, France) and the purity of nucleic acids was controlled by the ratio of absorbances at 260 nm and 280 nm (ratios between 1.8 and 2.2 were considered satisfactory). One microgram of total mRNA was used for reverse transcription with the iScript cDNA Synthesis Kit (Bio-Rad, Hercules, CA, USA) according to the following reaction protocol: 5 min at 25 °C, 1 h at 42 °C, 5 min at 85 °C. cDNA was amplified using the MESA GREEN qPCR MasterMix Plus for SYBR Assay w/fluorescein (Eurogentec, Liège, Belgium). All PCR reactions were performed on an Applied Biosystem Step One plus QPCR machine (Life Science Technologies, Overland Park, KS, USA). The primer sequences were: (*Abcd1:* forward 5′-gccaagttgtggatgag-3′ and reverse 5′-ttccgcagagtcgggataga-3′; *Abcd2:* forward 5′-tagaccgcatcctgcacagc-3′ and reverse 5′-ctccttcgccatcgaattgt-3′; *Abcd3:* forward 5′-ctgggcgtgaaatgactagattg-3′ and reverse 5′-cttctcctgttgtgacaccattg-3′; *Acox1:* forward 5′-gcccaactgtgacttccatt-3′ and reverse 5′-ggcatgtaacccgtagcact-3′; *Mfp2:* forward 5′-aggggacttcaagggaattgg-3′ and reverse 5′-gcctgcttcaactgaatcgtaa-3′. Thermal cycling conditions were as follows: activation of DNA polymerase at 95 °C for 10 min, followed by 40 cycles of amplification at 95 °C for 15 s, 60 °C for 30 s, and 72 °C for 30 s, followed by a melting curve analysis to control for the absence of non-specific products. Gene expression was quantified using cycle to threshold (*C*_t_) values and normalized by the *36B4* reference gene (forward 5′-atctgcttggagcccacat-3′ and reverse 5′-gcgacctggaagtccaacta-3′). The quantitative expression of *Abcd1*, *Abcd2*, *Abcd3*, *Acox1*, and *Mfp2* was determined as fold induction of the control.

### 3.12. Statistical Analyses

The experimental data represent the mean ± standard deviation. Statistical analyses were performed using SPSS 18.0 software (IBM Corporation, Armonk, NY, USA). The Mann–Whitney U test was used to compare the different groups, and data were considered statistically different at a *p*-value of 0.05 or less.

## 4. Conclusions

Our data support the hypothesis that 7KC, which is mainly formed endogenously by cholesterol auto-oxidation in the tissues and body fluids of patients subjected to oxidative stress, and which is increased in the plasma, the cerebrospinal fluid and the tissues of patients with neurodegenerative diseases (MS, X-ALD, Niemann–Pick disease) [21,22,23], could contribute to neurodegeneration via the induction of microglial dysfunction. Indeed, 7KC triggers mitochondrial and peroxisomal dysfunction in murine microglial BV-2 cells, two events that are known to promote neurodegeneration. In addition, our data show for the first time that tocopherols (α- and γ-tocopherol) and oleic acid, which are major compounds of the Mediterranean diet, but not Trolox, not only impair 7KC-induced mitochondrial dysfunction but also peroxisomal dysfunction (biogenesis evaluated with Abcd3 protein expression, and function, especially peroxisomal β-oxidation, evaluated with Abcd1, Abcd2, Acox1 and Mfp2 mRNAs levels). These data also suggest that eating habits could affect the occurrence of neurodegenerative diseases and enhance the interest of using natural compounds (tocopherols, polyphenols, fatty acids, etc.) to prevent and/or treat neurodegenerative diseases [120].

## Figures and Tables

**Table 1:** Fatty acid and tocopherol content of olive oils.

	Olive Oils		
	Morocco	Spain	Tunisia
Fatty acids (mg/g of total lipids)			
C12:0	0	0	0
C14:0	0	0	0
C15:0	0	0	0
C16:0	96.00 ± 0	102.00 ± 0.58	198.00 ± 6.51
C16:1 n-7	5.00 ± 0	7.00 ± 0	29.00 ± 1.00
C16:1 n-9	2.00 ± 0	1.00 ± 0	1.00 ± 0
C17:0	0	0	0
C18:0	30.70 ± 1.53	38.30 ± 1.53	24.70 ± 1.53
C18:1 trans	12.70 ± 3.51	14.00 ± 1.00	10.00 ± 2.00
C18:1n-9 (oleic acid)	664.00 ± 4.00 (2.354 ± 0.014 M)	745.00 ± 4.00 (2.641 ± 0.014 M)	471.00 ± 15.00 (1.670 ± 0.053 M)
C18:1 n-7	19.00 ± 0	22.00 ± 0	38.00 ± 1.00
C18:2 n-6 cis trans	0	0	0
C18:2 n-6 trans cis	0	0	0
C18:2 n-6	129.00 ± 3.00	36.00 ± 0	190.00 ± 3.51
C20:0	3.00 ± 0	4.00 ± 0	4.00 ± 0
C20:1 n-9	3.00 ± 0	2.00 ± 0	2.00 ± 0
C18:3 n-3	8.67 ± 0.58	6.00 ± 0	7.33 ± 0.58
C20:2 n-6	0	0	0
C22:0	1.00 ± 0	1.00 ± 0	1.00 ± 0
C22:1 n-9	0	0	0
C24:0	0	0	0
C24:1 n-9	0	0	0
Conjugated C18:3	0	0	0
Tocopherols (mg/kg of oil)			
α-tocopherol	30 ± 2 (69.605 ± 4.640 µM)	76 ± 2 (176.334 ± 4.640 µM)	112 ± 3 (259.860 ± 6.960 µM)
γ-tocopherol	5 ± 1 (11.990 ± 2.398 µM)	22 ± 1 (52.757 ± 2.398 µM)	9 ± 2 (21.582 ± 2.398 µM)
δ-tocopherol	0	0	0
Ratio [(α-tocopherol)/(γ-tocopherol)]	6	3.5	12.5

The molecular weights used to calculate the concentrations are the following: oleic acid (OA: 282 g/mol), α-tocopherol (431 g/mol), and γ-tocopherol (417 g/mol).

**Table 2:** Evaluation of the antioxidant properties of α-tocopherol, γ-tocopherol, Trolox, and oleic acid with FRAP, DPPH, and KRL tests.

Assays for Estimating Antioxidant Activity	Antioxidant Activity (Trolox Equivalent)		
	α-tocopherol	γ-tocopherol	Oleic Acid (OA)
FRAP	0.69 ± 0.06	1.86 ± 0.11	0.02 ± 0.01
DPPH	0.52 ± 0.05	0.86 ± 0.07	0.08 ± 0.01
KRL	0.94 ± 0.01	1.31 ± 0.13	ND

Data are presented in Trolox Equivalent (TE): one mole of α-tocopherol, γ-tocopherol, and oleic acid (OA) is equivalent to X mole (values shown in the Table) of Trolox. Data shown are mean of three independent experiments realized in triplicate. ND: not determined.

## Supplementary Materials

Supplementary materials can be found at www.mdpi.com/1422-0067/17/10/1973/s1.
