# Supplementary material for: Protective Effects of α-Tocopherol, γ-Tocopherol and Oleic Acid, Three Compounds of Olive Oils, and No Effect of Trolox, on 7-Ketocholesterol-Induced Mitochondrial and Peroxisomal Dysfunction in Microglial BV-2 Cells
Source: Int J Mol Sci. 2016 Nov 25;17(12):1973. doi: 10.3390/ijms17121973 (PMC5187773; doi:10.3390/ijms17121973)
Supplement: Supplementary file 1 [file ijms-17-01973-s001.pdf]

Supplementary Materials: Protective Effects of α-Tocopherol, γ-Tocopherol and Oleic Acid, Three Compounds of Olive Oils, and No Effect of Trolox, on 7-Ketocholesterol-Induced Mitochondrial and Peroxisomal Dysfunction in Microglial BV-2 Cells

Meryam Debbabi, Thomas Nury, Amira Zarrouk, Nadia Mekahli, Maryem Bezine, Randa Sghaier, Stéphane Grégoire, Lucy Martine, Philippe Durand, Emmanuelle Camus, Anne Vejux, Aymen Jabrane, Lionel Bretillon, Michel Prost, Thibault Moreau, Sofien Ben Ammou, Mohamed Hammami and Gérard Lizard

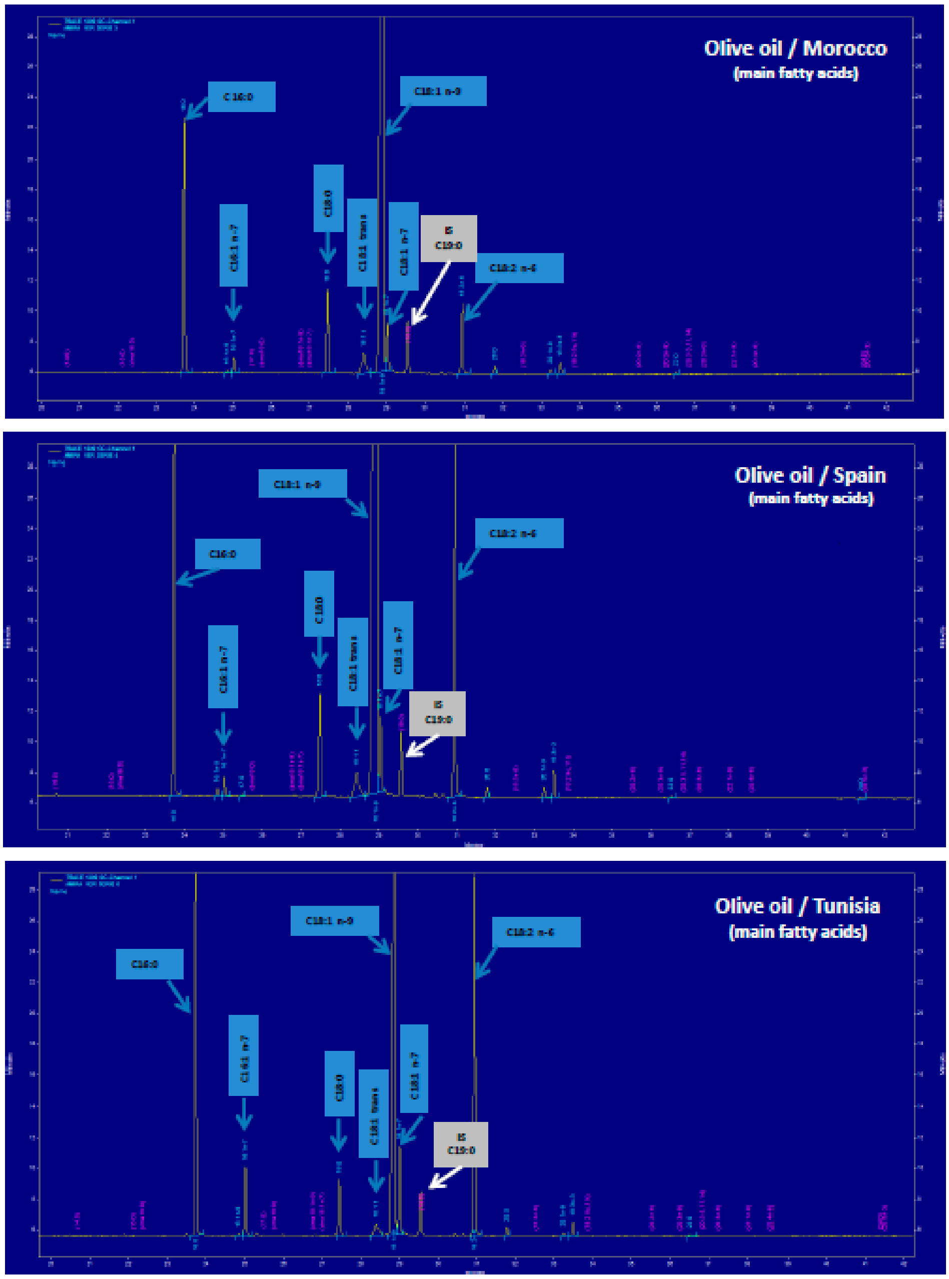

Figure S1. Debbabi M et al. Is means internal standard.

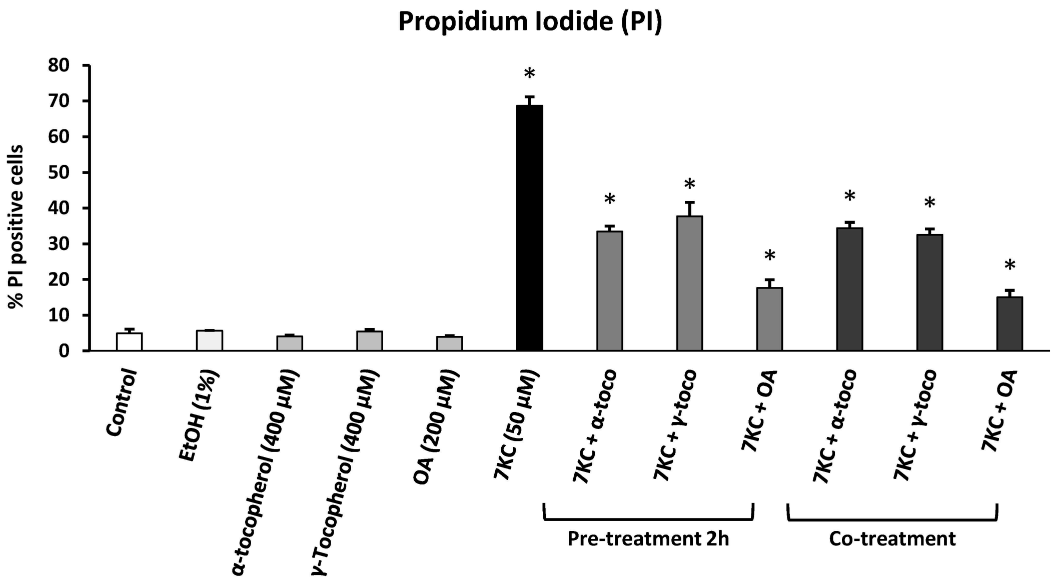

Figure S2. Debbabi M et al.
